# Patient Satisfaction and Visual Performance in Patients with Ocular Pathology after Bilateral Implantation of a New Extended Depth of Focus Intraocular Lens

**DOI:** 10.1155/2022/4659309

**Published:** 2022-04-28

**Authors:** Laureano A. Rementería-Capelo, Pilar Lorente, Virginia Carrillo, José M. Sánchez-Pina, Javier Ruiz-Alcocer, Inés Contreras

**Affiliations:** ^1^Clínica Rementería, Madrid, Spain; ^2^Optics and Optometry Department, Universidad Complutense de Madrid, Madrid, Spain; ^3^Hospital Universitario Ramón y Cajal Madrid, Instituto Ramón y Cajal de Investigaciones Sanitarias (IRYCIS), Madrid, Spain

## Abstract

**Aim:**

To evaluate visual results and patient-perceived outcomes in patients with ocular pathologies implanted with a new extended depth-of-focus intraocular lens (IOL).

**Methods:**

Patients with ocular pathology undergoing cataract surgery and bilaterally implanted with Vivity® IOLs were evaluated three months after surgery. The control group included patients with no ocular pathologies. Binocular defocus curves, corrected and uncorrected mono- and binocular distance visual acuity (DVA), and binocular contrast sensitivity were measured. Patients completed the Catquest-9SF questionnaire and reported on dysphotopsia and their need for spectacle-correction.

**Results:**

Twenty-five patients were included in each group. Monocular uncorrected DVA was better in the control group (−0.01 ± 0.07) compared with the study group (0.03 ± 0.08), *p*=0.027. There were no other statistically significant differences in DVA, with an uncorrected binocular acuity of −0.06 ± 0.06 for the control group and −0.05 ± 0.06 for the study group. Binocular defocus curves were similar for both groups and there were no differences in contrast sensitivity values. Pooling the refractive results, 96% of eyes were within ±0.50 D of target refraction. Seventy percent of patients in the control group reported no halos, compared with 40% in the study group, *p*=0.047. In both groups, 40% of patients reported being completely spectacle-independent, with the other 60% requiring glasses for near vision always or often. All patients reported being fairly or very satisfied with their vision.

**Conclusion:**

Initial results of visual function after Vivity implantation in patients with ocular pathologies are encouraging, with high patient satisfaction and few difficulties for daily activities.

## 1. Introduction 

Patients undergoing cataract surgery often seek to be spectacle-independent. Multifocal intraocular lenses (IOLs) were designed to address this expectation, providing functional visual acuity for far, intermediate, and near distances, and most patients implanted with these lenses do not require spectacle correction for their daily activities [1]. However, multifocal lenses have been associated with contrast sensitivity reduction and increased photic phenomena, such as halos and glare [1, 2]. These problems may reduce patient satisfaction with their vision in spite of an excellent visual acuity. In fact, a recent statement of the European Society of Cataract & Refractive Surgeons on functional vision suggested that the achievement of a certain distance visual acuity threshold is not always related to the patients' self-assessed vision improvement [3].

Since our current activities often require good intermediate vision, for example, for using smartphones, tablets, or desktop computers, extended depth of focus (EDOF), IOLs were developed to deliver an extended range of vision, which would cover distance and intermediate vision with good visual quality and a decreased risk of unwanted side effects such as photic phenomena [4]. These lenses may also be an option for healthy patients with potential risks of ocular pathologies. Different technologies have been developed to achieve the distribution of light throughout an elongated and continuous area of focus, the most popular of which include both diffractive designs and nondiffractive models based on optical aberrations. The TECNIS Symfony IOL (Johnson & Johnson Surgical Vision, Inc, Santa Ana, Calif.) uses a biconvex design, an anterior aspheric surface, and a posterior achromatic diffractive surface with an echelette design to obtain the extended depth of focus effect [5]. The AT-Lara (Carl Zeiss Meditec, Jena, Germany) has a patented smooth surface design with shallower angles, with a biconvex aspheric, achromatic diffractive anterior surface [6]. Among the nondiffractive models is the Mini-Well IOL (SIFI MedTech Srl, Catania, Italy), which has three zones: a central zone with positive spherical aberration, a middle zone with negative spherical aberration, and an outer monofocal zone. Combined, these zones provide EDOF through asphericity. The TECNIS Eyhance (Johnson & Johnson, New Brunswick, NJ) uses an aspheric design to increase negative aberration [4,6]. The AcrySof IQ Vivity IOL uses wavefront-shaping technology to increase the depth of focus. In the pivotal FDA approval trial, the Vivity IOL achieved a mean corrected distance visual acuity (CDVA) noninferior to the monofocal control IOL and a superior mean distance-corrected intermediate visual acuity (DCIVA), with 72.9% of eyes achieving a DCIVA of 0.20 logMAR or better six months after surgery. Distance corrected near visual acuity (DCNVA) was also better than the control IOL, with 40.2% of eyes achieving 0.30 logMAR or better. Rates of reports of severe or very bothersome visual disturbances were low, less than 4% and 3% for the Vivity and the monofocal control IOL, respectively. As a drawback, the Vivity IOL was associated with a reduction in monocular mesopic contrast sensitivity compared to the monofocal control IOL [7].

Several studies have recently reported outcomes in patients implanted with the Vivity IOL in a clinical setting [8–11]. However, these reports exclude patients with ocular pathologies. Clinicians are usually reluctant to implant trifocal IOLs in these patients because of the impact the decrease in contrast sensitivity and the dependence on ambient lighting might have on visual function. Since the Vivity lens is not based on diffractive technology, it might be a viable option for patients with ocular pathologies, who wish to reduce spectacle-dependance after cataract surgery. The purpose of this study was to compare visual results and patient-perceived outcomes between a group of patients with prior ocular pathologies implanted with the Vivity IOL and a control group of healthy subjects.

## 2. Methods

This prospective study included patients with ocular pathology who were implanted with a Vivity IOL. Age- and gender-matched healthy patients who had been implanted with a Vivity IOL during the same period were invited to participate in order to form a control group.

Prior to surgery, patients in our clinic undergo a comprehensive evaluation including CDVA, slit-lamp examination, tonometry, corneal topography (Pentacam HR model 70,900, Oculus, Germany), endothelial cell count (with a CEM-530 specular biomicroscope, NIDEK CO, LDT, Japan), biometry (including pupilometry) with the IOLMaster 700 (Carl Zeiss Meditec AG, Jena, Germany), fundus evaluation after pharmacological mydriasis, and optic nerve head and macular optical coherence examination with spectral domain technology (Cirrus HD-OCT 5000, Carl Zeiss Meditec AG, Germany). Toric IOLs are usually recommended in patients with 0.8 diopter (D) or more preoperative corneal astigmatism. Candidates for toric IOL implantation are also explored with the VERION Image Guided System (Alcon Laboratories, USA).

Based on the patient´s pathologies, lifestyle, and expectations, the attending ophthalmologist recommends a specific type of IOL. Patients are informed of the advantages of each type of IOL and the potential problems, including the need for spectacle correction for certain activities, loss of contrast, and the need for sufficient light for adequate visual function. Patients were only asked to participate in the study at the one-month visit after surgical intervention; therefore, there was no influence on the IOL recommendation for each patient. The study was performed in accordance with the tenets of the Declaration of Helsinki and its amendments; it was reviewed and approved by our reference ethics committee. Patients were informed of the nature of the study and signed a consent form before inclusion.

Inclusion criteria for the study group were the presence of any ocular pathology other than age-related cataracts and bilateral implantation of a Vivity IOL. Inclusion criteria for the control group were the absence of any ocular pathology other than age-related cataracts and bilateral implantation of a Vivity IOL. Patients with intra- or postoperative complications were excluded from the study.

Cataract surgery was performed under topical and intracameral anesthesia through a 2.2 mm clear corneal incision, with a standard stop and chop sutureless phacoemulsification technique and intracameral cefuroxime. If the IOL was toric, implantation was guided with the Verion Vision System.

Postoperative treatment consisted of a fixed combination of dexamethasone and tobramycin three times daily for one week, combined with bromfenac one drop twice daily for three weeks. Second eye surgery was performed between two and seven days after the first eye. Patients were seen on the day following surgery, as well as one week, one month, and three months after second eye surgery. Results presented herein are those of the three-month visit.

At the three-month visit, patients underwent an extensive evaluation, including binocular defocus curve, corrected and uncorrected mono- and binocular visual acuity for distance (4 meters), and binocular contrast sensitivity using the functional acuity contrast test (Test SV-1000) of the CC-100 HW 5.0 Series system, Topcon. Patients also completed the Catquest-9SF questionnaire and questions evaluating the presence of dysphotopsia and their need for spectacle-correction for different distances. This protocol is similar to the one we employed to study outcomes in a prior study reporting on outcomes in patients receiving trifocal lenses and another extended depth of focus IOL [5].

In addition, patients underwent intraocular pressure measurement, slit-lamp evaluation, fundus evaluation after pharmacological mydriasis, corneal topography, and OCT evaluation.

The AcrySof IQ Vivity lens is a single-piece, biconvex, hydrophobic, acrylic foldable IOL, UV absorbing and with a blue light filter. It has an optical zone of 6.0 mm, and an overall diameter of 13 mm. On the anterior surface of the central 2.2 mm of the optic, it has a modified structure based on X-wave technology, which provides the extended depth of focus effect and is not based on refractive technology. The optic also induces a negative spherical aberration. The X-wave or wavefront-shaping technology leads to two physical phenomena, which may be described as “stretch and shift.” The first raised plateau “stretches” the wavefront, creating an extended focus area. The second element “shifts” the wavefront so that no light is lost.

Biometric IOL power calculations were performed with the Barrett formula. The power of the toric IOLS and their implantation axis were calculated with the online calculators provided by the IOL's manufacturer. Spherical power was taken from the IOL Master report (the first IOL with the lowest negative spherical equivalent); the corneal values employed were those provided by the IOL Master. All eyes were targeted for emmetropia.

### 2.1. Statistical Analysis

Statistical analysis was performed using SPSS version 26 software (IBM statistics). Due to the low number of patients in each group, nonparametric tests were employed for comparisons, either Mann-Whitney or Chi square. The software version automatically adjusted the *p* values according to the Bonferroni correction for multiple comparisons. Statistical significance was set at a *p* value <0.05. Visual acuity results are provided in LogMAR scale.

## 3. Results

The study group included 25 patients, and the control group included 25 patients. Fourteen men were included in each group (56%), and the age was similar in both groups: 71.84 ± 5.62 years, range 60 to 83, in the study group, and 71.36 years ± 5.61, range 60 to 81, in the control group (*p*=0.669). Twenty-nine eyes (58%) in the study group received a toric IOL compared with 26 eyes (52%) in the control group, *p*=0.546. Patients in the ocular pathology group included six patients with glaucoma (24%); four patients with cornea guttata (16%); three patients with dry age-related macular degeneration (12%); two patients each with ocular hypertension (8%), amblyopia (8%), and corneal leucoma (8%); one patient with epiretinal membrane (4%), macular telangiectasia (4%), lagophthalmos due to facial nerve palsy (4%), homonymous hemianopia (4%), previous LASIK surgery (4%), and daltonism (4%). Prior refractive surgery is not strictly an ocular pathology; however, as in the case of daltonism, it might interfere with the IOL mechanism for creating an extended depth of focus effect, and therefore, they were included in the ocular pathology group. In four of the patients with glaucoma and two of the patients with ocular hypertension, cataract surgery was combined with I-Stent implantation. There were no statistically significant differences in preoperative characteristics: CDVA, pupil size, keratometric cylinder, axial length, intraocular lens power, and target spherical equivalent (Table 1). Postoperative distance visual acuity and refraction, prediction errors, and incidence of dysphotopsias are recorded in Table 2. There was only a statistically significant difference in monocular uncorrected distance visual acuity (UCDVA): −0.01 ± 0.07 for the control group versus 0.03 ± 0.08 for the study group, *p*=0.027. However, this difference would not be clinically relevant. To calculate the prediction errors, the predicted postoperative refraction was subtracted from the measured spherical equivalent refraction. Thus, a positive prediction error indicates a refractive outcome that was more hyperopic than predicted. Pooling the refractive results for both groups, 96% of eyes were within ±0.50 D of target refraction and 100% within ±0.75 D.

**Figure 1:**
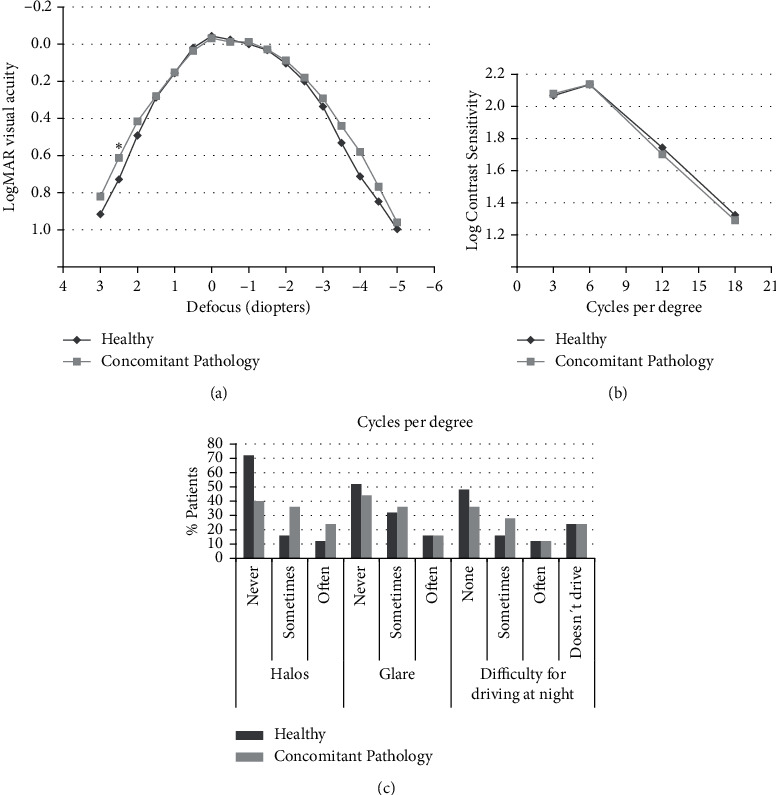
(a) Defocus curves for the study group (concomitant pathology) and the control group (healthy subjects). The asterisk (*∗*) marks the only defocus value, in which there was a statistically significant difference between groups (+2.50 diopters, *p*=0.032). (b) Contrast sensitivity. There were no statistically significant differences between groups. (c) Prevalence of halos, glare, and difficulty for driving at night.

Figure 1(a) shows the binocular defocus curves for both groups: there was only a statistically significant difference for +2.5 D defocus although, again, the difference would not be clinically relevant.  Figure 1(b) shows contrast sensitivity values, which were almost identical for both groups.  Figure 1(c) shows patient reported presence of halos, glare, and difficulty for driving at night. Seventy percent of patients in the control group reported no halos, compared with 40% in the study group; the difference was statistically significant (*p*=0.027). Patients in the control group also reported less glare and less difficulty for driving at night, although the differences were not statistically significant (*p*=0.248 and *p*=0.194, respectively). Only one patient reported always experiencing halos: a 61-year-old lady with cornea guttata. This patient has been followed up for one year after surgery, and she now reports no halos or glare. No patient required spectacle correction for distance vision. Only two patients (8%) in the control group reported using spectacle-correction for intermediate distance, sometimes. For near vision, in the control group, three patients (12%) reported always requiring correction, 12 patients (48%), sometimes, and 10 patients (40%), never. In the study group, one patient (4%) always required spectacles for near vision, 14 patients (56%), sometimes, and 10 patients (40%), never. There were no statistically significant differences.

**Figure 2:**
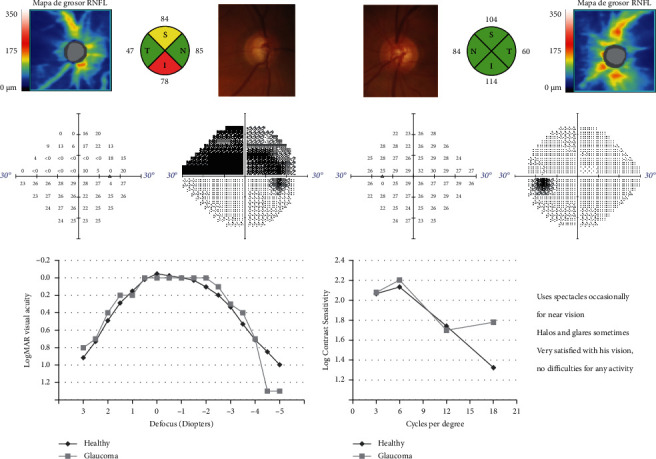
Visual fields, optic nerve optical coherence tomography, and visual results after bilateral Vivity intraocular lens implantation combined with I-Stent injection in a 74-year-old man with bilateral glaucoma with severe visual field damage in the right eye. The combined results of the healthy control group are shown for comparison.

The results of the visual satisfaction questionnaire are reflected in Table 3. Interestingly, patients in the study group reported a higher satisfaction with their visual performance than patients in the control group (*p*=0.016), and patients in the control group reported a greater difficulty for reading newspapers (*p*=0.030). There were no other statistically significant differences between groups. All patients stated they would undergo surgery again with the same type of IOL.  Figure 2 shows the visual fields and visual outcomes of a 73-year-old patient with glaucoma.

## 4. Discussion

The aim of this study was to report the initial outcomes of patients with coexisting ocular pathologies after cataract surgery and bilateral implantation of a Vivity IOL. The visual acuity results were very good, with both groups achieving a mean binocular uncorrected visual acuity better than 0.0 logMAR (Snellen equivalent 20/20). Statistically significant differences were only found for uncorrected monocular acuity and for the +2.5 D value of the defocus curve, although these differences would not be clinically relevant. This is probably because the patients in the ocular pathology group did not have advanced forms of disease. Curiously, patients with ocular pathologies had a higher satisfaction than healthy patients. This might be due to a lower expectation for improved outcomes after surgery; in the preoperative discussion, the attending ophthalmologist probably highlighted the possible drawbacks of the IOL and the uncertain outcomes. It might also be because patients with some visual loss due to maculopathy or glaucoma might be more tolerant to image defocus and might adapt more rapidly [2]. The difference in visual expectations might also explain the difference in the reported difficulty for reading text in newspapers: patients with ocular pathologies might be used to having more difficulties. Another possible explanation is that patients with ocular pathologies reported a greater use of glasses for near vision, which would reduce their difficulties for reading small print. It might also be due to the small sample size, which might magnify small differences.

There have been few reports on the outcomes of the Vivity IOL up to date. Arrigo et al. reported on the refractive outcome; far, intermediate, and near vision and quality of vision score in 54 healthy patients [9]. Gundersen and Potvin studied the effects on the binocular defocus curve in 40 healthy patients by simulating myopia in the nondominant eye [8] and a group from the Netherlands reported the refractive outcomes, defocus curves, spectacle independence, photic phenomena, and Catquest-9SF questionnaires in 22 healthy patients targeted for monovision [11]. Kohnen et al. studied these same outcome measures in a group of 16 healthy patients targeted for emmetropia [10].

Kohnen et al. reported a monocular UCDVA and CDVA of 0.12 ± 0.14 logMAR and 0.01 ± 0.05 logMAR, respectively [10], while the group from the Netherlands found a CDVA of −0.04 ± 0.11 logMAR in the dominant eye [11]. Arrigo et al. reported a monocular UCDVA and CDVA of 0.1 ± 0.04 and 0.0 ± 0.03, respectively [9]. Corrected monocular visual acuity in our study was similar to these results, for both the healthy group (−0.01 ± 0.06) and the study group (0.00 ± 0.06). Uncorrected monocular visual acuity in our study was better than reported by Kohnen et al. [10], possibly due to the lower postoperative spherical equivalent in our patients (−0.04 ± 0.17 D in the control group and −0.07 ± 0.19 D in the study group) compared with the one in their study: −0.16 ± 0.356 D with 90.6% of eyes within ±0.50 D of target refraction, compared with 96% of eyes in our study. The Netherland group reported a postoperative spherical equivalent in the dominant eye of 0.11 ± 0.31 D [11] and Arrigo et al. of −0.1 ± 0.2 D [9].

Regarding binocular function, Kohnen et al. reported a binocular UCDVA and CDVA of 0.01 ± 0.05 and −0.02 ± 0.07, respectively, [10] and the Netherland group with their monovision approach, −0.07 ± 0.10 and −0.10 ± 0.08, respectively [11]. Again, this compares favourably with the uncorrected values of −0.06 ± 0.06 for the control group and −0.05 ± 0.06 for the study group and corrected values of −0.06 ± 0.06 for both groups in our study.

The binocular defocus curves we found were very similar for patients with and without ocular pathology, with a LogMAR acuity of 0.10 or better between −2.00 and + 0.50 defocus. Visual acuity for −1.50 D defocus was 0.03 for both groups, and for −2.50 D defocus, it was 0.18 for the study group and 0.2 for the control group. This compares favourably with Kohnen et al. s study, in which LogMAR acuity was 0.10 and 0.38 for −1.50 D and −2.50 D defocus, respectively [10]. In fact, it seems to be similar also to the results reported in the study of mini-monovision, in which binocular defocus curves showed a visual acuity greater than 0.10 logMAR in the range from −2.0 D to +0.5 D, with values of 0.01 and 0.24 logMAR for −1.50 D and −2.50 D defocus, respectively. [11].

In our study, we found that patients reported a higher incidence of halos and glare than in other reports on the Vivity IOL: 72% and 52% of patients in the control group reported no halos or glare, respectively, versus 40% and 44% in the study group, whilst Kohnen et al. found that 75% reported no halos and 75% no glare [10]. In the Netherland study, the percentage of patients who experienced no halos, glare, or starbursts were 91%, 91%, and 100%, respectively [11]. Arrigo et al. reported that only 30% and 33% of patients reported halos and glare, respectively [9]. The differences between our study and others might be because, in our case, patients were actively asked about the incidence of dysphotopsias, and this has been shown to increase the rate of reporting [12].

In our study, 40% of patients in both groups reported never using glasses for near distance. This rate is similar to that reported by Kohnen et al. (38%) [10] and with the mini-monovision strategy (between 24 and 38% depending on lighting conditions) [11].

Visual satisfaction is difficult to compare between studies due to the different questionnaires employed. The Netherland group using the Catquest found that the percentage of subjects who responded to be “very satisfied” and “fairly/very satisfied” with their vision was 68% and 91%, respectively [11]. In our study, 52% of the healthy group and 84% of the study group reported being “very satisfied” and all patients reported being “fairly/very satisfied.” Overall, 84% and 72% reported that their vision did not give them difficulties in their daily lives.

The main limitation of our study is the wide range of ocular pathologies that the patients included in the study group presented. This, together with the low number of patients included overall, as well as for each pathology, means that it is difficult to extrapolate results. Studies focusing on each individual pathology, with a higher number of patients, would be necessary to be able to determine from which type of IOL they would benefit the most. Another limitation is the short follow-up period. It is known that neuroadaptation plays a major role concerning optical phenomena, with a significant decrease in difficulties with time. An example is the patient with cornea guttata who reported a constant presence of glare three months after surgery and no dysphotopsias one year after the procedure.

In summary, this is to the best of our knowledge the first report on the implantation of the Vivity IOL in patients with coexisting ocular pathologies. Results are encouraging, with a very high patient satisfaction, few difficulties for daily activities, and relatively high spectacle independence for near vision.

## Figures and Tables

**Table 1:** Preoperative characteristics of the eyes included in the study. mm: millimeters; D: diopters.

	Healthy	Coexisting pathology	*P*
LogMAR corrected visual acuity	0.19 ± 0.28	0.15 ± 0.17	0.828
	1.30 to 0.00	0.70 to 0.00	

Pupil (mm)	2.69 ± 0.58	2.86 ± 0.62	0.205
	1.77 to 4.30	1.83 to 4.52	

K1 (D)	43.06 ± 0.96	43.16 ± 1.87	0.603
	40.80 to 44.80	38.40 to 47.70	

K2 (D)	43.82 ± 0.97	43.87 ± 1.95	0.836
	41.60 to 45.40	39.10 to 48.30	

Keratometric cylinder (D)	0.75 ± 0.45	0.70 ± 0.41	0.529
	0.00 to 1.70	0.10 to 1.80	

Axial length (mm)	24.01 ± 0.79	23.96 ± 1.11	0.796
	22.58 to 26.43	21.15 to 26.67	

Intraocular lens power (D)	20.05 ± 2.41	20.20 ± 2.65	0.722
	15.00 to 24.00	15.00 to 25.00	

Target spherical equivalent (D)	−0.18 ± 0.15	−0.13 ± 0.16	0.291
	−0.54 to 0.10	−0.43 to 0.30	

**Table 2:** Postoperative LogMAR distance visual acuity, refractive status, and prediction error. Values provided are mean (standard deviation) and range. *P* value is from the Kruskall-Wallis test.

	Healthy	Coexisting pathology	*P*
Binocular uncorrected distance visual acuity	−0.06 ± 0.06	−0.05 ± 0.06	0.604
	0.00 to −0.20	0.10 to −0.20	

Binocular corrected distance visual acuity	−0.06 ± 0.06	−0.06 ± 0.06	0.887
	0.00 to −0.20	0.00 to −0.20	

Monocular uncorrected distance visual acuity	−0.01 ± 0.07	0.03 ± 0.08	0.027
	0.30 to −0.20	0.30 to −0.10	

Monocular corrected distance visual acuity	−0.01 ± 0.06	0.00 ± 0.06	0.300
	0.20 to −0.20	0.20 to −0.10	

Sphere (diopters)	−0.02 ± 0.08	−0.06 ± 0.16	0.137
	−0.50 to 0.00	−0.75 to 0.00	

Cylinder (diopters)	−0.06 ± 0.26	−0.04 ± 0.16	0.993
	−1.75 to 0.00	−0–75 to 0.00	

Spherical equivalent (diopters)	−0.04 ± 0.17	−0.07 ± 0.19	0.229
	−0.87 to 0.00	−0.75 to 0.00	

Mean prediction error (diopters)	0.14 ± 0.20	0.06 ± 0.23	0.148
	−0.56 to 0.54	−0.52 to 0.43	

Mean absolute prediction error (diopters)	0.20 ± 0.14	0.20 ± 0.13	0.748
	0.01 to 0.56	0.01 to 0.52	

**Table 3:** Results of the Catquest questionnaire. For the seven difficulty items (questions 3 to 7), the question is: do you have difficulty with the following activities because of your vision? The points assigned for statistical analysis to the possible answers (except for question 2) are as follows: very great difficulties, 1; great difficulties, 2; some difficulties, 3; no difficulties, 4. For question 2, possible answers are very dissatisfied, 1; rather dissatisfied, 2; fairly satisfied, 3; very satisfied, 4.

	Healthy	Coexisting pathology	*P*
1. Do you experience that your present vision gives you difficulties in any way in your daily life?	3.84 ± 0.37	3.78 ± 0.42	0.615
	3 to 4	3 to 4	

2. Are you satisfied or dissatisfied with your present vision?	3.52 ± 0.51	3.84 ± 0.37	0.016
	3 to 4	3 to 4	

3. Difficulties for reading text newspaper	3.28 ± 0.61	3.60 ± 0.71	0.030
	2 to 4	3 to 4	

4. Difficulties for recognizing faces	3.92 ± 0.28	4.00 ± 0	0.153
	3 to 4	4	

5. Difficulties for seeing prices	3.72 ± 0.46	3.84 ± 0.37	0.311
	3 to 4	3 to 4	

6. Difficulties for walking	3.96 ± 0.20	3.96 ± 0.20	1.000
	3 to 4	3 to 4	

7. Difficulties performing handicraft	3.63 ± 0.50	3.52 ± 0.71	0.777
	3 to 4	3 to 4	

8. Difficulties reading text on TV	3.92 ± 0.28	3.80 ± 0.41	0.226
	3 to 4	3 to 4	

9. Difficulties for preferred hobby	3.84 ± 0.37	3.92 ± 0.28	0.389
	3 to 4	3 to 4	

## Data Availability

Data are available from the corresponding author upon reasonable request.
